# Supplementary material for: Double Strike in Chronic Lymphocytic Leukemia—The Combination of BTK and BCL2 Inhibitors in Actual and Future Clinical Practice
Source: Int J Mol Sci. 2025 Mar 29;26(7):3193. doi: 10.3390/ijms26073193 (PMC11989886; doi:10.3390/ijms26073193)
Supplement: Supplementary file 1 [file ijms-26-03193-s001.zip › ijms-3482203-supplementary.pdf]

Supplementary Material S1. Ongoing clinical trials examining the safety and efficacy of BTKi and BCL2i combinations, excluding venetoclax/ibrutinib.

Clinical trials investigating Venetoclax and Acalabrutinib combination				
**Clinical Trial Number**	**Indication**	**Trial status**	**Phase**	Description
NCT03868722	Newly diagnosed CLL	Active, recruiting	II	The study will investigate the reduction of the risk of infection – and thus mortality – after three months of combination therapy of venetoclax and acalabrutinib administered as a preventive treatment to patients with newly diagnosed CLL .
NCT04523428	R/R CLL	Active, recruiting	II	The study will evaluate the efficacy of venetoclax and acalabrutinib combination in relapsed patients after first line of venetoclax and anti-CD20 mAb treatment.
NCT03580928	First-line treatment of CLL	Active, not recruiting	II	The study assesses the the safety and efficacy of acalabrutinib, venetoclax and obinutuzumab combination.
NCT03128879	High-risk CLL	Active, recruiting	II	The study investigates the therapeutic efficacy of venetoclax added to ibrutinib or acalabrutinib in patients with detectable and high-risk CLL after receiving ibrutinib or acalabrutinib for at least 12 months.
NCT05197192	First-line treatment for high-risk CLL	Active, recruiting	III	The research will evaluate if a triple combination of acalabrutinib, obinutuzumab and venetoclax (GAVe) leads to prolonged progression-free survival, compared to using obinutuzumab and venetoclax combination (GVe).
NCT03836261	First-line treatment of CLL	Active, not recruiting	III	The study aims to assess the efficacy and safety of acalabrutinib plus venetoclax and acalabrutinib plus venetoclax plus obinutuzumab in comparison to chemoimmunotherapy.
NCT05336812	Treatment-naïve CLL	Active, recruiting	II	The study investigates if combining acalabrutinib with venetoclax or obinutuzumab is effective at reducing tumor size in CLL patients who have not received any prior treatment.
NCT05388006	Richter transformati on from CLL/SLL	Active, recruiting	II	The investigation of efficacy of acalabrutinib, venetoclax and durvalumbab combination in patients with Richter transformation from CLL or SLL.
NCT06524375	First-line treatment of CLL	Active, recruiting	II	The study will assess whether adding venetoclax to patients treated with cBTKi (ibrutinib, acalabrutinib or zanubrutinib) for the treatment-naïve CLL can lead to deep durable remissions of uMRD.
NCT04169737	High-risk/R/R CLL/SLL	Active, recruiting	II	The study will assess the efficacy of acalabrutinib and venetoclax with or without early obinutuzumab in patients with high-risk/recurrent/refractory CLL or SLL.
NCT05057494	First-line treatment of CLL/SLL	Active, not recruiting	III	The study will compare the efficacy of acalabrutinib plus venetoclax (AV) versus venetoclax plus obinutuzumab (VO) in first-line CLL or SLL.
NCT04941716	R/R CLL/SLL	Active, recruiting	II	The study will assess the efficacy of acalabrutinib and venetoclax combination in R/R CLL or SLL patients.
**Clinical trials investigating Venetoclax and Zanubrutinib combination**				
NCT05168930	R/R CLL/SLL	Active, recruiting	II	The study will assess three groups of patients who relapsed on the prior treatment: A) without prior BTKi or BCL2i treatment; B) with prior BTKi or BCL2i treatment; C) relapsing during therapy with covalent BTKi but not zanubrutinib

NCT04515238	R/R CLL	Active, not recruiting	II	The study will evaluate the efficacy and safety of bendamustine followed by synergistic therapy of obinutuzumab, zanubrutinib, and venetoclax in R/R CLL patients.
NCT03824483	First-line treatment for CLL/SLL or MCL	Active, recruiting	II	The research investigates the MRD response in patients previously untreated for CLL/SLL or MCL during the combination therapy of zanubrutinib, obinutuzumab, and venetoclax.
NCT05650723	First-line treatment for CLL/SLL	Active, recruiting	II	The study evaluates the possibility of the addition of obinutuzumab in MRD-positive patients receiving the combination therapy of zanubrutinib and venetoclax.
NCT05478512	First-line treatment for high-risk CLL	Active, recruiting	II	The investigation of the venetoclax obinutuzumab combination followed by venetoclax in MRD-negative patients and zanubrutinib and venetoclax in patients with residual disease.
**Clinical trials investigating Venetoclax and Pirtobrutinib combination**				
NCT05317936	CLL/SLL	Active, not recruiting	II	The study will assess the efficacy of pirtobrutinib and venetoclax combination in patients who did not achieve uMRD after 12 cycles of venetoclax.
NCT04965493	R/R CLL/SLL	Active, not recruiting	III	This research is a comparison of efficacy and safety of pirtobrutinib addition to venetoclax and rituximab vs venetoclax and rituximab alone. This study enrolls patients, who received at least one prior BTKi.
NCT05536349	First-line treatment for CLL/SLL or Richter Transformati on	Active, recruiting	II	The study will examine the rationale for pirtobrutinib, venetoclax, and obinutuzumab combination in treatment-naive CLL or RT patients.
NCT06466122	CLL/SLL relapsing in covalent BTKi	Active, recruiting	II	The study evaluating the combination of pirtobrutinib and venetoclax in patients progressing on covalent BTKi.
NCT05677919	First-line treatment for CLL	Active, recruiting	II	The investigation of pirtobrutinib-venetoclax combination in treatment-naive CLL guided by uMDR .
**Clinical trials investigating Sonrotoclax (BGB-11417) and Zanubrutinib combination**				
NCT06073821	First-line treatment for CLL	Active, recruiting	III	The comparison between sonrotoclax and zanubrutinib vs venetoclax and obinutuzumab for first-line CLL treatment
NCT06697184	First-line treatment for CLL	Not yet recruiting	I, II	The investigation of the safety of dose ramp-up schedule of sonrotoclax; initial zanubrutinib fixed-time monotherapy followed by zanubrutinib sonrotoclax combination at ramp-up schedules
NCT06367374	First-line treatment for CLL/SLL	Not yet recruiting	II	The investigation of sonrotoclax plus zanubrutinib with MRD-driven treatment duration in patients with previously untreated Chronic Lymphocytic Leukemia (CLL) or Small Lymphocytic Lymphoma (SLL)
NCT06637501	First-line treatment for CLL/SLL	Active, recruiting	II	The Comparison of the efficacy and safety of Sonrotoclax Combined With Zanubrutinib and Zanubrutinib Monotherapy
NCT04277637	Mature B-Cell malignancie s including	Active, recruiting	I	The investigation of the safety and tolerability of sonrotoclax monotherapy and its combination with zanubrutinib and obinutuzumab

	CLL/SLL cohort, both treatment-naive and relapsed/refractory to at least one prior treatment			
**Clinical trials investigating Lisaftoclax (APG-2575) and BTKi combination**				
NCT06104566	R/R CLL/SLL	Active, recruiting	III	The study will investigate the efficacy and safety of lisaftoclax in combination with BTK inhibitors in CLL/SLL patients who were previously treated with BTK inhibitors.
NCT06319456	First-line treatment for CLL/SLL	Active, recruiting	III	The study will investigate the efficacy and safety of lisaftoclax in combination with acalabrutinib versus immunochemotherapy in patients with newly diagnosed CLL/SLL.
NCT04494503	R/R CLL/SLL	Active, recruiting	Ib/II	The study will assess the safety, pharmacokinetic and efficacy of lisaftoclax single and in combination with ibrutinib or rituximab in R/R CLL/SLL patients.
